# Clinical and Immunological Profile of Mixed Connective Tissue Disease and a Comparison of Four Diagnostic Criteria

**DOI:** 10.1155/2020/9692030

**Published:** 2020-01-29

**Authors:** Kevin John John, Mohammad Sadiq, Tina George, Karthik Gunasekaran, Nirmal Francis, Ebenezer Rajadurai, Thambu David Sudarsanam

**Affiliations:** Department of Medicine, Christian Medical College, Vellore 632004, India

## Abstract

Mixed connective tissue disease (MCTD) was initially described as a chronic immune-mediated disease with overlapping features of systemic lupus erythematosus, scleroderma, and polymyositis. We conducted a cross-sectional study to describe the clinical and immunological profile of patients with MCTD and to compare the four diagnostic criteria, namely, Sharp, Kasukawa, Alarcón-Segovia, and Khan criteria. A total of 291 patients who were admitted from June 2007 to June 2017 and fulfilled the inclusion criteria were included in the study. A clinical diagnosis of MCTD was made in 111 patients, of whom 103 (92.8%) were women. The mean age at presentation was 39.3 years (SD ± 11.6). The most common organ systems that were involved were musculoskeletal system (95.5%), skin and mucosa (78.4%), and the gastrointestinal and hepatobiliary systems (56%). The maximum sensitivity was for the Kasukawa criteria with a sensitivity of 77.5% (95% CI 68.4-84.6) and specificity of 92.2% (95% CI 87-95.5). The Kahn criteria and Alarcón-Segovia criteria had the maximum specificity; the Alarcón-Segovia criteria had a sensitivity of 69.4% (95% CI 59.8-77.6) and a specificity of 99.4% (95% CI 96.5-99.9), while the Kahn criteria had a sensitivity of 52.3% (95% CI 42.6-61.7) and a specificity of 99.4% (95% CI 96.5-99.9). The sensitivity and specificity of Sharp criteria were 57.7% (95% CI 47.9-66.87) and 90% (95% CI 84.4-93.8), respectively.

## 1. Introduction

Mixed connective tissue disease (MCTD) was initially described as a chronic immune-mediated disease with overlapping features of systemic lupus erythematosus (SLE), scleroderma, and polymyositis [1]. The characteristic feature of this disease, which separates it as a distinct clinical entity, is the presence of antibodies against U1 ribonucleoprotein (RNP) complex [2]. A study conducted in Norway estimated the incidence and prevalence of MCTD to be 2.1 per million per year and 3.8 per 1,00,000 adults, respectively. The female to male ratio was 3.3, and the mean age at diagnosis was 37.9 years [3]. Sharp and his colleagues had initially described MCTD as a mild disease with good response to steroids and a favourable outcome. [1] Hajas et al. have recently reported a 5, 10, and 15-year survival rate of 98%, 96%, and 88%, respectively, which correlates with Sharp's observations [4].

Connective tissue disorders can present with a plethora of symptoms and signs. The diagnosis of these disorders is made based on specific criteria. These criteria are updated as new evidence emerges. The major connective tissue diseases are SLE, systemic sclerosis, polymyositis, dermatomyositis, rheumatoid arthritis, and primary Sjogren's syndrome.

However, many patients have clinical features which overlap between these diseases. Also, the patient may recruit new symptoms and signs, and the clinical picture may change over time. As a result, the clinical features of a patient who presents with an “undifferentiated” connective tissue disease, may evolve into those of one of the diseases mentioned above.

Because of these reasons, there has been considerable debate on whether MCTD should be considered a distinct connective tissue disease. Apart from the presence of high titers of anti-U-1 RNP antibody, patients with MCTD have been found to have a higher incidence of Raynaud's phenomenon and pulmonary hypertension [2, 5]. They have less severe renal involvement and have a better overall prognosis [4, 6]. The phenotypic stability of MCTD has also been established [7]. Hence, it is now accepted that MCTD is a distinct entity [8, 9]. Over the years, there have been various efforts to create a standardized diagnostic criterion for MCTD. The four criteria that have stood the test of time are the Sharp's criteria, the Kasukawa diagnostic criteria, the Alarcón-Segovia criteria, and the Kahn's criteria [10–13]. Although comparisons among the criteria are limited, the Alarcón-Segovia and Kahn's criteria have demonstrated the best sensitivity and specificity [14].

There has been a substantial body of research on various aspects of MCTD from around the world. However, there is a dearth of data from India. Here, we describe the largest cohort of patients from India affected by MCTD. We have described the clinical and immunological profile of the disease and compared the sensitivity and specificity of the diagnostic criteria.

## 2. Objective

The objectives of this study were to describe the clinical and immunological profile of patients with MCTD and compare the four diagnostic criteria, namely, Sharp, Kasukawa, Alarcón-Segovia, and Khan criteria (Table 1).

## 3. Materials and Methods

### 3.1. Study Design

This was a cross-sectional study which was approved by the Institutional Review Board (IRB) before its commencement. The investigation protocol complied with Good Clinical Practices and the Declaration of Helsinki. The results are reported in accordance with the STROBE (Strengthening the Reporting of Observational Studies in Epidemiology) guidelines for reporting observational studies [16].

### 3.2. Setting

This study was conducted in the Christian Medical College and Hospital, Vellore, a 2858 bedded university teaching institute in South India. All patients who were admitted to the Department of Medicine from June 2007 to June 2017 were included in the study.

### 3.3. Participants

All patients admitted to the department of medicine with a clinical diagnosis of MCTD and overlap or undifferentiated connective tissue disease during the study period were included in the study. Patients with other diagnosis were excluded. Data were retrieved from the electronic medical database.

### 3.4. Variables

Organ system involvement and symptoms at presentation were recorded. Immunological profile was assessed in each case from laboratory data. The four diagnostic criteria mentioned above were applied in all cases.

### 3.5. Statistical Methods

The patient data were extracted from electronic medical records and analysis was done using SPSS 25 (IBM, Armonk, NY). All categorical baseline data were described using numbers and percentages. Continuous data were described using mean and standard deviation. Sensitivity and specificity of the four diagnostic criteria were calculated against a clinical diagnosis of mixed connective tissue disease as the reference standard.

## 4. Results

Out of the 291 patients who were admitted from June 2007 to June 2017 with a diagnosis of MCTD and undifferentiated or overlap connective tissue disease, a clinical diagnosis of MCTD had been made in 111 patients.

### 4.1. Baseline Characteristics and Clinical Features

Of the 111 patients, 103 (92.8%) were women. The mean age at presentation was 39.3 years (SD ± 11.6). The most common organ systems that were involved were musculoskeletal system (95.5%), skin and mucosa (78.4%), and the gastrointestinal and hepatobiliary systems (56%) (Table 2). The most common symptoms were arthritis (69.4%) and Raynaud's phenomenon (66.7%). Interstitial lung disease (ILD) (38.7%) and sclerodactyly (36.9%) were also common. The most common pattern of neuropathy was trigeminal neuropathy, and the most common pattern of glomerulonephritis was membranous, closely followed by mesangioproliferative glomerulonephritis. Anti-U1-RNP antibodies were positive in all patients (Table 3). ANA was positive in 89.2% of the patients, and the speckled pattern was the most common. Thirteen patients (11.7%) died in the hospital during the period covered by the study.

### 4.2. Comparison of Diagnostic Criteria

The maximum sensitivity was for the Kasukawa criteria with a sensitivity of 77.5% (95% CI 68.4-84.6) and specificity of 92.2% (95% CI 87-95.5). Both Kahn criteria and Alarcón-Segovia criteria had equal specificity while the latter was found to be more sensitive. The Alarcón-Segovia criteria had a sensitivity of 69.4% (95% CI 59.8-77.6) and a specificity of 99.4% (95% CI 96.5-99.9) while the Kahn criteria had a sensitivity of 52.3% (95% CI 42.6-61.7) and a specificity of 99.4% (95% CI 96.5-99.9). The sensitivity and specificity of Sharp criteria were 57.7% (95% CI 47.9-66.9) and 90% (95% CI 84.4-93.8), respectively.

## 5. Discussion

From its initial description in 1972, our understanding of mixed connective tissue disease has come a long way. It is now recognized that MCTD is a distinct entity with unique clinical features, response to therapy, and prognosis. Recent studies by Reiseter *et al*. have established the stability of MCTD phenotype [7]. Here, we have described the largest cohort of patients with MCTD in India.

The mean age at presentation of our patients was similar to that found in previously published cohorts. Our study supports a female preponderance in MCTD. However, the number of women was much higher in our study compared to data from Norway [3]. This may be due to differences in the expression of disease, between males and females in the Indian population. However, since ours is a tertiary care referral center, referral bias is also possible. Large-scale community-based studies may be required to clarify this observation. A large number of patients present with nonspecific symptoms like fever and fatigue. Often, the diagnosis of MCTD is initially missed and later made correctly when the disease evolves. Arthritis and Raynaud's phenomenon were widespread in our cohort. Sclerodactyly, ILD, and myositis were also common. Hence, the combination of arthritis, Raynaud's phenomenon, ILD, sclerodactyly, and myositis in a patient with constitutional symptoms of fever and fatigue should alert the physician to consider MCTD in the list of differentials.

Global data suggests that deforming arthritis may be seen in approximately 60% of patients with MCTD. Rheumatoid factor was positive in about 70% of the patients in our study. [17, 18] In this study, although the overall prevalence of arthritis was nearly the same (69.4%), only 6 had deforming arthritis (5.4%). We have also observed that a lesser proportion of patients had a positive rheumatoid factor (22.5%) when compared to other cohorts. Whether this is causation or association is unclear at this point, and further research into the pathophysiology of the disease may clarify this in the future.

Pulmonary artery hypertension (PAH) is a major cause of mortality in MCTD and is probably underdiagnosed. The prevalence of PAH in MCTD has been estimated differently by different studies. A Norwegian multicenter study estimated a prevalence of 3.4%, while others have observed a prevalence of 14-18% [4, 19, 20]. We found a low prevalence of PAH in our patients (8.1%). We observed a 38.7% prevalence of ILD, which was similar to what has been reported elsewhere (41%) [21]. The Norwegian study identified male gender, elevated anti-U-1 RNP titer, and absence of arthritis as predictors of ILD. We found no such association. Renal involvement was less common compared to western data (17% versus 40%) [6]. Majority of the patients had membranous glomerulonephritis. Oesophagal dysfunction was also less common in our patients (28.8%) [22]. These factors point to fundamental differences between the MCTD phenotype in Indian patients when compared with those discussed in the existing literature.

As expected, all the patients had elevated anti-U-1 RNP antibody, and ANA was positive in a majority of patients. The speckled pattern was most common. However, it should be noted that anti-U-1 RNP may be positive in other connective tissue diseases also. In one study, these antibodies were positive in 20-40% of patients with SLE, 2-14% of patients with systemic sclerosis, and 6-9% of patients with myositis [9]. More than half the patients had a direct Coombs test positive hemolytic anemia (56.7%). Prevalence of anti-dsDNA, rheumatoid factor, anti-Ro/SS-A, and anti-La/SS-B was much lower. It must be noted that anti-dsDNA and anti-Smith antibodies are only transiently positive in MCTD [23]. However, if dsDNA or anti-Smith is the dominant and persistent autoantibody, a diagnosis of SLE must be strongly considered over MCTD as these autoantibodies are highly specific for the former diagnosis. In the present study, the antibodies reported were those at initial presentation and it is not known whether the same antibodies were the predominant ones on follow-up. This remains a limitation of the present study. It must also be noted that the presence of these autoantibodies at admission may bias against the Sharp criteria, where anti-Smith is an exclusion criteria.

The Alarcón-Segovia and Khan criteria are considered the best to diagnose MCTD [24]. However, our data suggest that the Kasukawa criteria is the most sensitive, while both Alarcón-Segovia and Khan criteria have the maximum specificity. Hence, we would recommend that the Kasukawa criteria be used to screen patients and rule out the disease, while either the Alarcón-Segovia or Khan criteria be used to rule in the disease.

### 5.1. Strength and Limitations

The strength of our study was the large sample size, given the rarity of this disease. The limitation of this study is its retrospective study design. This can be overcome by well-designed, prospective studies in the future.

### 5.2. Generalizability

This study was conducted in a tertiary care hospital, which may affect the generalizability of these results. One way of overcoming this limitation is by performing community-based studies, but it may not be feasible considering the rarity of this disease. Also, all participants in this study were from India. The manifestation of disease may vary depending on ethnicity and race, which needs to be kept in mind while applying the results of this study.

## 6. Conclusions

The most common presenting features of MCTD were arthritis, Raynaud's phenomenon, ILD, and sclerodactyly. Hence, the constellation of these findings in a patient with constitutional symptoms of fever and fatigue should alert the physician to consider MCTD in the list of differentials. Anti-U1-RNP antibodies were positive in all patients and ANA with a speckled pattern was positive in most of the patients. The expression of MCTD in the Indian population was different from that in the patients described in other studies in that there is a more marked female preponderance, arthritis was less commonly deforming, and the prevalence of PAH and renal involvement was lesser. The most sensitive diagnostic criterion was the Kasukawa criteria, while the most specific criteria were the Kahn criteria and the Alacron-Segovia criteria. Hence, the Kasukawa criteria should be used to rule out the disease, while either the Alarcón-Segovia or Kahn criteria should be used to rule in the disease.

## Figures and Tables

**Table 1:** Diagnostic criteria for mixed connective tissue disorder [15].

Sharp	Major criteria	Minor criteria	Diagnosis
	(1) Myositis (2) Pulmonary involvement: (a) Diffusion capacity < 70% of normal values (b) Pulmonary hypertension (c) Proliferative vascular lesions on lung biopsy (3) Raynaud's phenomenon or esophageal hypomotility (4) Swollen hands (5) Anti-ENA Ab N 1 : 10,000 and anti-U1 RNP Ab positive and anti-Sm negative	(1) Alopecia (2) Leukopenia (3) Anemia (4) Pleuritis (5) Pericarditis (6) Arthritis (7) Trigeminal neuropathy (8) Malar rash (9) Thrombocytopenia (10) Mild myositis (11) History of swollen hands	At least 4 major criteria plus anti-U1-RNP Ab titer of at least 1 : 4000 or two major criteria from among criteria 1, 2, and 3 plus 2 minor criteria plus anti-U1-RNP Ab titer of at least 1 : 1000 Exclusion criteria: positivity for anti-Sm Ab

Kasukawa	Common symptoms	Mixed symptoms	Diagnosis
	(1) Raynaud's phenomenon (2) Swollen fingers or hands anti-RNP Ab positive	(1) SLE-like symptoms: (a) Polyarthritis (b) Lymphadenopathy (c) Facial erythema (d) Pericarditis or pleuritis (e) Leukopenia or thrombocytopenia. (2) SSc-like findings: (a) Sclerodactyly (b) Pulmonary fibrosis, restrictive changes of lung, or reduced diffusion capacity (c) Hypomotility or dilatation of esophagus. (3) PM-like findings: (a) Muscle weakness (b) Elevated serum levels of muscle enzymes (CPK) (c) Myogenic pattern on EMG	At least one of common symptoms plus positivity for anti-RNP Ab plus one or more signs/symptoms of the mixed symptoms in at least two of the three disease categories

Alarcón-Segovia	Serological criteria	Clinical criteria	Diagnosis
	Anti-RNP Ab titer N 1 : 1000	(1) Edema in hands (2) Synovitis (3) Myositis (4) Raynaud's phenomenon (5) Acrosclerosis	Serological criteria plus at least 3 clinical criteria included either synovitis or myositis

Kahn	Serological criteria	Clinical criteria	Diagnosis
	Presence of high titer anti-RNP Ab corresponding to speckled ANA at titer ≥ 1 : 2000	(1) Raynaud's phenomenon (2) Synovitis (3) Myositis (4) Swollen fingers	Serological criteria plus Raynaud's phenomenon and at least two of the three following signs (synovitis, myositis, and swollen fingers)

Abbreviations: RNP: ribonucleoprotein; ENA: extractable nuclear antigen; Sm: Smith; SLE: systemic lupus erythematosus; SSc: systemic sclerosis; PM: polymyositis; CPK: creatine phosphokinase; EMG: electromyography; ANA: anti-nuclear antigen.

**Table 2:** Clinical features of mixed connective tissue disease.

Symptom	Number (%), *n* = 111
Central nervous system	20 (18)
Seizure	4 (3.6)
Headache	9 (8.1)
Neuropathy	10 (9)^∗^
Meningitis	3 (2.7)
Cardiovascular system	13 (11.3)
Myocarditis	2 (1.8)
Pericardial effusion	9 (8.1)
Cor pulmonale	1 (0.9)
Left ventricular failure	1 (0.9)
Respiratory system	55 (49.5)
Pleural effusion	7 (6.3)
Interstitial lung disease	43 (38.7)^◇^
Pulmonary arterial hypertension	9 (8.1)
Hematological system	24 (21.6)
Autoimmune hemolytic anemia	21 (18.9)
Immune thrombocytopenia	5 (4.5)
Gastrointestinal and hepatobiliary system	62 (56)
Hepatitis	19 (17.1)
Autoimmune hepatitis	11 (9.9)
Esophageal dysmotility	32 (28.8)
Genitourinary system	19 (17.1)
Glomerulonephritis	19 (17.1)^□^
Skin and mucosal system	87 (78.4)
Malar rash	27 (24.3)
Sicca symptoms	16 (14.4)
Mucosal ulcerations	34 (30.6)
Alopecia	30 (27)
Sclerodactyly	41 (36.9)
Raynaud's phenomenon	74 (66.7)
Digital gangrene	5 (4.5)
Musculoskeletal system	106 (95.5)
Arthritis^▲^	77 (69.4)
Myositis	42 (37.8)
Swollen fingers	22 (19.8)
Miscellaneous	
Fatigue	27 (24.3)
Lymphadenopathy	11 (9.9)
Fever at presentation	51 (45.9)

^∗^Four patients had trigeminal neuropathy. ^◇^All patients had predominant lower zone involvement. ^□^Seven patients had membranous glomerulonephritis. Six had mesangioproliferative glomerulonephritis, and 2 had focal segmental glomerulonephritis. ^▲^Six patients had deforming arthritis.

**Table 3:** Antibody profile of mixed connective tissue disease.

Antibody	Number (%), *n* = 111
Anti-U1RNP	111 (100)
Anti-nuclear antibody	99 (89.2)
Anti-Scl-70	2 (1.8)
Anti-Ro/SSA	12 (10.8)
Anti-La/SSB	5 (4.5)
Anti-double-stranded DNA	30 (27)
Anti-Smith	4 (3.6)
Rheumatoid factor	25 (22.5)
Anticardiolipin	6 (5.4)
Lupus anticoagulant	13 (11.7)
Anti-Jo-1	2 (1.8)
Direct Coombs test	63 (56.7)

## Data Availability

The data used to support the findings of this study are available from the first author and corresponding author upon request.
